# Performance of CUN-BAE in Assessing Adiposity and Visceral Fat: A Validation Study Using Bioelectrical Impedance Analysis in a Mexican Population

**DOI:** 10.3390/diagnostics16121786

**Published:** 2026-06-10

**Authors:** Paulo César Gete Palacios, Alejandra Fabiola Vázquez López, Eduardo Salif Luna-Avila, Luis Angel López-Cruz, Manuel Ramón García-Sáenz, Emmanuel Valdez-Solis, Mario Alberto Santana-Mata, Claudia Ramírez-Rentería, Aldo Ferreira-Hermosillo

**Affiliations:** 1 Unidad de Investigación Médica en Enfermedades Endocrinas, Centro Médico Nacional Siglo XXI, Instituto Mexicano del Seguro Social, Mexico City 06720, Mexico; paulo.getepa@universidad.anahuac.mx (P.C.G.P.); ale.vazquez.1712@gmail.com (A.F.V.L.); esalif@gmail.com (E.S.L.-A.); langel251120@gmail.com (L.A.L.-C.); cramirez@endocrinologia.org.mx (C.R.-R.); 2 Servicio de Endocrinología, Hospital de Especialidades “Dr. Bernardo Sepúlveda”, Centro Médico Nacional Siglo XXI, Instituto Mexicano del Seguro Social, Mexico City 06720, Mexico; manuel.garcias@unam.edu; 3 Hospital Ángeles Tampico, Tampico 89330, Tamaulipas, Mexico; emmanuelmarinvaldezsolis@gmail.com; 4 Médica Bosco, Saltillo 25253, Coahuila, Mexico; santana.endocrinologia.md@gmail.com

**Keywords:** adiposity, body composition, body fat distribution, obesity, body mass index

## Abstract

**Background/Objectives**: This cross-sectional study aimed to validate the Clínica Universidad de Navarra-Body Adiposity Estimator (CUN-BAE) against bioelectrical impedance analysis (BIA) in 204 adults. **Methods**: We evaluated CUN-BAE and body mass index (BMI) as continuous predictors of body fat percentage, total fat mass, and visceral fat. **Results**: BMI showed stronger correlations with visceral fat (r = 0.941 vs. 0.850) and total fat mass (r = 0.968 vs. 0.828),while CUN-BAE showed a slightly stronger correlation with body fat percentage (r = 0.918 vs. 0.884), the parameter it was designed to estimate. CUN-BAE displayed only a weak correlation with muscle mass (r = 0.153), supporting its specificity for adiposity assessment, whereas BMI showed a moderate correlation with muscle mass (r = 0.551), reflecting its weight-dependent nature. **Conclusions**: Overall, BMI remains a practical marker of absolute fat mass and visceral adiposity, while CUN-BAE may provide a more refined estimate of relative body fat percentage. Tool selection should be guided by the clinical objective and interpreted as complementary rather than competing measures.

## 1. Introduction

For decades, the body mass index (BMI), which is calculated as weight in kilograms divided by height in meters squared (kg/m^2^), has served as the cornerstone of obesity classification in clinical practice, public health surveys and epidemiological research. Its widespread adoption is largely attributable to its simplicity, non-invasive nature, and low cost. However, the fundamental limitation of BMI lies in its inability to differentiate between fat mass and fat-free mass, primarily composed of skeletal muscle, bone and water [1,2]. This limitation leads to significant clinical misclassifications. For instance, individuals with high muscle mass, such as athletes or physically active individuals, may be incorrectly classified as overweight or obese despite having a low percentage of body fat [3]. Conversely, and perhaps more insidiously, individuals with a normal BMI may possess a disproportionately high percentage of body fat and low muscle mass, a condition termed “normal weight obesity” or “metabolically obese normal weight”, which carries a significantly elevated risk of insulin resistance and cardiovascular morbidity [4,5].

This recognition has shifted the paradigm from a simple weight-centric view to a more nuanced appreciation of body composition. The distribution of adipose tissue represents a critical determinant of metabolic health. Subcutaneous adipose tissue (SAT), while contributing to total adiposity, is considered less metabolically harmful than visceral adipose tissue (VAT). VAT, the fat depot located within the abdominal cavity surrounding internal organs, is a highly active endocrine and immune organ [6,7]. It secretes a plethora of pro-inflammatory cytokines (e.g., TNF-a and IL-6), adipokines, and free fatty acids directly into the portal circulation, leading to systemic inflammation, hepatic insulin resistance and the development of atherogenic dyslipidemia [8,9,10].

The accurate quantification of VAT is therefore paramount for refined risk stratification. However, the gold-standard methods for its measurement, such as dual-energy X-ray absorptiometry (DEXA), computed tomography (CT) and magnetic resonance imaging (MRI), are expensive, involve radiation exposure (in the case of CT), and are not feasible for routine clinical use or large-scale screening [11]. Simultaneously, the importance of skeletal muscle mass is increasingly recognized. Muscle is the primary site for glucose disposal and a key regulator of resting metabolic rate. The age-related loss of muscle mass and function, known as sarcopenia, and its coexistence with obesity, are associated with disability, metabolic dysfunction, and increased mortality [12,13]. The inability of BMI to detect either low muscle mass or the sarcopenic obesity phenotype underscores its insufficiency as a standalone diagnostic tool.

In response to these limitations, several alternative anthropometric indices and equations have been developed to provide a more accurate estimation of body fat. Derived from a large Spanish cohort, the Clínica Universidad de Navarra-Body Adiposity Estimator (CUN-BAE) is a simple mathematical formula that incorporates age, sex, and BMI to estimate body fat percentage (%BF), addressing some of the key demographic variables that affect body composition. The original validation study by Gómez-Ambrosi et al. demonstrated its superiority over BMI in predicting actual %BF as measured by dual-energy X-ray absorptiometry (DEXA) [14].

While subsequent studies have consistently demonstrated CUN-BAE’s strong correlation with total adiposity, its relationship with body compartments remains less comprehensively explored. Therefore, this cross-sectional study aims to validate the CUN-BAE index against direct body composition measurements by BIA and to quantify its incremental value beyond BMI when estimating body fat percentage and visceral adiposity.

We also explore whether those indices relate to a functional sarcopenia screening score (SARC-F). The SARC-F questionnaire is a brief, self-reported screening tool developed to rapidly identify individuals at risk of sarcopenia through five functional domains. Its total score ranges from 0 to 10, and a score ≥ 4 has been proposed as suggestive of sarcopenia risk. Because sarcopenia diagnosis requires objective assessment of muscle strength, muscle mass, and muscle quality, SARC-F should be interpreted as a practical screening instrument rather than a definitive diagnostic tool. Therefore, in the present study, SARC-F was included as an exploratory secondary objective to evaluate whether adiposity-focused indices such as BMI and CUN-BAE are associated with functional sarcopenia risk [15,16]. 

## 2. Materials and Methods

### 2.1. Study Design and Participants

This was an observational, analytical, cross-sectional study with ambispective data collection. We included adult patients, men and women, with a diagnosis of obesity (body mass index ≥ 30 kg/m^2^) who attended the Hospital de Especialidades, Centro Médico Nacional Siglo XXI, Instituto Mexicano del Seguro Social, located in Mexico City, (Ethics Committee protocol code R-2017-3601-225), and who were under follow-up at this institution (at least three consecutive visits in the last year) with stable medication use (no changes in the last eight weeks). Patients were not included if they had incomplete clinical records or if they declined to participate in the study. Participants were recruited using a non-probabilistic consecutive sampling method among patients who met the eligibility criteria during the study period.

All participants underwent a comprehensive clinical history, including demographic data, medical background, lifestyle factors, and the presence of previously diagnosed metabolic comorbidities such as type 2 diabetes mellitus, hypertension, dyslipidemia, and obesity-related conditions. Data were collected through structured interviews and systematically corroborated with information extracted from each participant’s clinical records.

### 2.2. Anthropometric Assessment

Anthropometric assessment was performed by trained personnel following standardized protocols. Body composition and weight were evaluated using the InBody120 body composition analyzer (InBody Co., Ltd., Seoul, Republic of Korea), and height was measured using the stadiometer of a Detecto mechanical physician scale, model 2392 (Cardinal Scale Mfg. Co., Webb City, MO, USA), with participants barefoot and wearing light clothing. Waist and hip circumferences were measured using a non-elastic measuring tape using an SECA 201 circumference measuring tape (SECA gmbh & co. kg, Hamburg, Germany) at standardized anatomical landmarks. The body mass index (BMI) was calculated as weight in kilograms divided by height in meters squared (kg/m^2^) and was analyzed as a continuous variable [17].

### 2.3. CUN-BAE Calculation

To improve the estimation of body fat beyond BMI, the CUN-BAE was calculated using the established equation that incorporates age, sex and BMI: %BF = −44.988 + (0.503 × age) + (10.689 × sex) + (3.172 × BMI) − (0.026 × BMI^2^) + (0.181 × BMI × sex) − (0.02 × BMI × age) − (0.005 × BMI^2^ × sex) + (0.00021 × BMI^2^ × age), where sex is coded as zero for male and one for female. This index provides an indirect estimation of body fat percentage and has demonstrated a strong correlation with bioimpedance and dual-energy X-ray absorptiometry (DXA), as well as predictive value for cardiometabolic risk in adult populations [14].

### 2.4. Functional Sarcopenia Screening

Sarcopenia risk was assessed using the SARC-F questionnaire, a validated screening tool composed of five domains: strength, assistance in walking, rising from a chair, climbing stairs, and falls. Each domain was scored from zero to two, yielding a total score ranging from zero to ten. A score of ≥4 points was considered indicative of probable sarcopenia and functional impairment [15].

### 2.5. Body Composition Assessment by Bioelectrical Impedance Analysis

Body composition was evaluated using the InBody 120 system (model BPM040S12F07) based on multifrequency bioelectrical impedance analysis (BIA). This technology measures impedance at multiple frequencies to differentiate intracellular and extracellular water and provides segmental analysis of body compartments. The parameters obtained included total body fat mass (in kilograms and percentage), skeletal muscle mass (in kilograms), visceral fat level, and segmental lean mass distribution. The InBody device is non-invasive, radiation-free, and has demonstrated acceptable agreement with reference techniques such as DXA, making it suitable for clinical and research settings [18,19,20,21].

### 2.6. Variables and Outcomes

All bioelectrical impedance measurements were performed in the morning by trained personnel following manufacturer guidelines. Participants were instructed to avoid heavy meals and vigorous physical activity prior to the measurement, to abstain from alcohol consumption for 48 h, and to empty their bladder immediately prior to assessment. Measurements were conducted in light clothing, barefoot, and without metallic objects. Participants remained standing for at least 5 min before analysis to ensure fluid stabilization.

The analyzed variables included quantitative measures (age, BMI, CUN-BAE [%], fat mass, skeletal muscle mass, visceral fat, and waist circumference) and qualitative measures (sex, presence of metabolic comorbidities, and sarcopenia risk defined as SARC-F 4).

### 2.7. Statistical Analysis

Data distribution was assessed using normality tests. Continuous variables were expressed as mean ± standard deviation or median and interquartile range, as appropriate. Primary analyses treated adiposity indices (BMI and CUN-BAE) as continuous predictors. Associations between BIA-derived parameters (body fat percentage, total fat mass, visceral fat level and skeletal muscle mass) were assessed using Pearson or Spearman correlation coefficients. To quantify incremental validity beyond BMI, multivariable linear regression models were fitted to each BIA outcome, comparing a base model including BMI with an expanded model including CUN-BAE (or equivalently BMI plus age and sex terms represented in the CUN-BAE formula); model fit was compared using changes in R2 and information criteria where applicable. As an exploratory analysis, the associations between adiposity indices and probable sarcopenia (SARC-F 4) were evaluated using logistic regression. Statistical significance was defined as *p* < 0.05. The analyses were performed with SPSS version 25.

Methodological Rationale: The use of CUN-BAE allows for a more accurate characterization of adiposity, particularly in populations where BMI alone may underestimate fat mass. The SARC-F questionnaire enables rapid identification of individuals at risk of sarcopenia in clinical practice, while InBody bioimpedance analysis provides a practical and validated approach for assessing muscle and fat compartments, supporting its application in metabolic and functional research.

## 3. Results

This analytical cross-sectional study included 204 participants, whose baseline characteristics are detailed in Table 1. The population consisted predominantly of women, 65% (*n* = 133), with a mean age of 41 ± 12 years.

**Figure 1:**
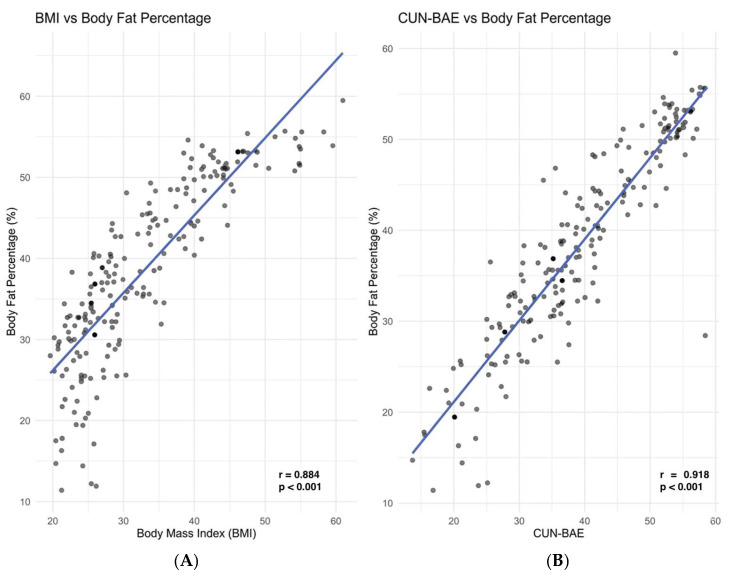
Association between BMI and CUN-BAE with body fat percentage. Scatterplots illustrating the linear relationship between (**A**) body mass index (BMI) and body fat percentage and (**B**) CUN-BAE and body fat percentage as measured by bioelectrical impedance analysis. Solid lines represent linear regression fits. CUN-BAE demonstrates a stronger correlation with body fat percentage, consistent with its algorithmic design to estimate adiposity. Pearson’s correlation test was used to assess the associations shown in this figure.

**Figure 2:**
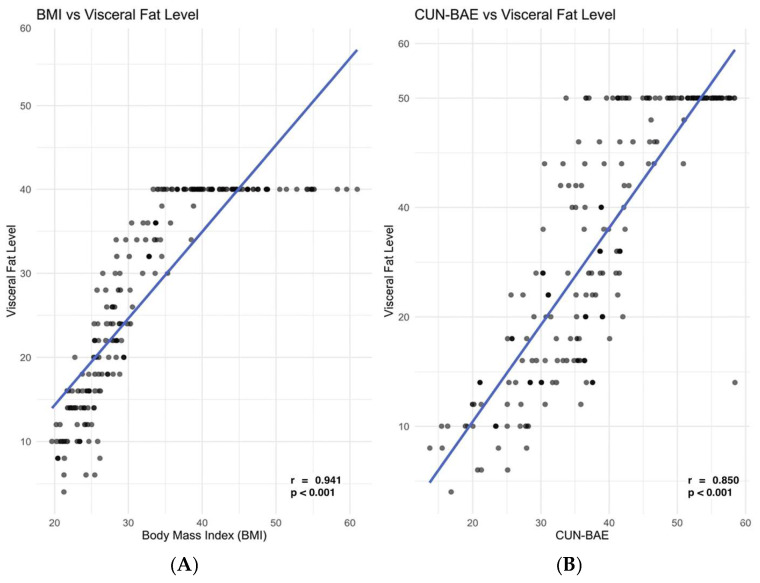
Association between BMI and CUN-BAE with visceral fat level. Scatterplots illustrating the relationship between (**A**) BMI and visceral fat level and (**B**) CUN-BAE and visceral fat level as assessed by bioelectrical impedance analysis. Both indices show strong positive associations with visceral adiposity, although the relationship reflects an indirect estimation rather than direct imaging-based quantification. Pearson’s correlation test was used to assess the associations shown in this figure.

The results showed highly significant correlations between CUN-BAE and adiposity parameters, specifically, we found strong correlations with body fat percentage (r = 0.918, *p* < 0.001), visceral fat (r = 0.850, *p* < 0.001), and total fat mass (r = 0.828, *p* < 0.001). In contrast, a weak correlation with muscle mass was observed (r = 0.153, *p* = 0.029). Meanwhile, BMI also showed strong correlations with body fat percentage (r = 0.884, *p* < 0.001), visceral fat (r = 0.941, *p* < 0.001), and total fat mass (r = 0.968, *p* < 0.001), as well as a moderate correlation with muscle mass (r = 0.551, *p* < 0.001) (Figure 1 and Figure 2).

We assessed these correlations by sex. In the male population, CUN-BAE was strongly correlated with body fat percentage (r = 0.935, *p* < 0.001), visceral fat (r = 0.916, *p* < 0.001), total fat mass (r = 0.953, *p* < 0.001), and moderately correlated with muscle mass (r = 0.655, *p* < 0.001). Meanwhile, in the female population, CUN-BAE was strongly correlated with body fat percentage (r = 0.896, *p* < 0.001), visceral fat (r = 0.889, *p* < 0.001), total fat mass (r = 0.911, *p* < 0.001) and muscle mass (r = 0.904, *p* < 0.001). Regarding BMI, in the male population, it was strongly correlated with body fat percentage (r = 0.910, *p* < 0.001), visceral fat (r = 0.843, *p* < 0.001), total fat mass (r = 0.981, *p* < 0.001), and moderately correlated with muscle mass (r = 0.695, *p* < 0.001), while in the female population it was strongly correlated with body fat percentage (r = 0.925, *p* < 0.001), visceral fat (r = 0.884, *p* < 0.001), total fat mass (r = 0.979, *p* < 0.001), and moderately correlated with muscle mass (r = 0.769, *p* < 0.001).

A regression analysis was performed to further characterize the relationships of CUN-BAE with different adiposity parameters, adjusting for clinically relevant variables including glycemic status, hypertension, hypothyroidism, lung disease, smoking, alcohol, exercise, diet and nutritional counseling. CUN-BAE remained independently associated with total body fat percentage and total fat mass in adjusted models (Table 2).

After adjustment for the same variables, BMI also remained associated with total body fat percentage. However, this association was positively influenced only by diet (β = 3.631, 95% CI: 0.577–6.680, *p* = 0.020). Regarding the association of CUN-BAE with visceral fat percentage, when adjusting for glycemic status, muscle mass, hypertension, lung disease, ideal weight, and diet, it demonstrated a moderate relationship. This association was significantly influenced by ideal weight and muscle mass. Specifically, muscle mass was negatively associated with the relationship between CUN-BAE and visceral fat (β = −1.268, CI: −1.700–−0.836, *p* < 0.001), revealing a significant modulating effect of muscle mass on this relationship. Meanwhile, ideal weight (β = 0.554, CI: 0.293–0.814, *p* < 0.001) was positively associated with this relationship. These findings suggest that baseline body characteristics significantly influence the index’s accuracy for estimating this specific adipose compartment. Finally, after adjusting for confounding variables, BMI also remained associated with visceral fat percentage and was positively influenced by diet (β = 2.343, CI: 0.870–3.821, *p* = 0.002).

**Figure 3:**
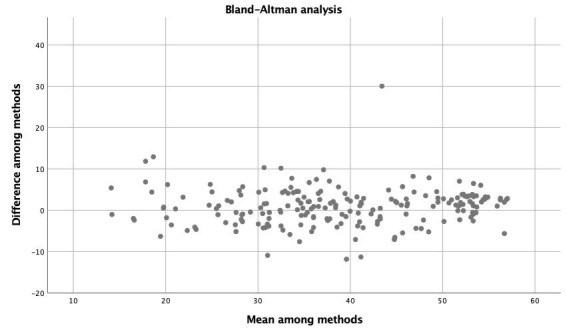
Bland–Altman plot comparing CUN-BAE-estimated body fat percentage with BIA-derived body fat percentage.

Bland–Altman analysis comparing CUN-BAE-estimated body fat percentage with BIA-derived body fat percentage showed a small positive mean bias of 0.93 percentage points, indicating a slight average overestimation by CUN-BAE relative to BIA. The 95% limits of agreement ranged from −7.89 to 9.75 percentage points, suggesting that although the average difference between methods was small, individual-level discrepancies may occur. Therefore, CUN-BAE showed acceptable agreement for estimating body fat percentage at the group level, but it should not be considered fully interchangeable with BIA for precise individual body composition assessment (Figure 3).

The SARC-F score mode value was 0, with minimum and maximum values ranging from 0 to 5. Only one participant met the SARC-F ≥ 4 cutoff for probable sarcopenia risk. Given the extremely low prevalence of probable sarcopenia risk according to SARC-F, further association analyses with BMI or CUN-BAE were not considered statistically reliable.

The results demonstrate that CUN-BAE is an excellent indicator of total and visceral adiposity but has limited capacity for assessing muscle mass, validating its clinical utility for metabolic stratification but not for sarcopenia evaluation.

## 4. Discussion

The escalating global prevalence of obesity has reached pandemic proportions, establishing itself as one of the most significant public health challenges of the 21st century. Current epidemiological data from the WHO (World Health Organization) indicates that obesity rates have tripled since 1975, with over 650 million adults worldwide currently living with obesity [22]. This trend continues to be concerning given the well-established association between excess adiposity and the spectrum of cardiometabolic diseases, which include type 2 diabetes (T2D), hypertension, dyslipidemia, cardiovascular disease and certain types of cancer [23]. The economic burden on healthcare systems is substantial, driven by both direct medical costs and indirect costs related to productivity losses, making the effective diagnosis and management of obesity a critical priority [24].

The present study provides a comprehensive validation of the CUN-BAE index in a Mexican cohort, using bioelectrical impedance analysis (BIA) as a reference method, and offers a nuanced comparative analysis with body mass index (BMI). Our findings confirm that both indices are valuable but serve distinct clinical purposes, delineating a context-dependent paradigm for their use.

### 4.1. Validation and Specificity of the CUN-BAE Index

The strong associations between CUN-BAE and adiposity parameters remained robust in multivariate analyses adjusted for key confounders, including glycemic status and hypertension. CUN-BAE persisted as an independent predictor of both total body fat percentage (β = 0.882) and total fat mass (β = 0.433), confirming its clinical utility extends beyond a reflection of underlying metabolic conditions. Crucially, its weak correlation with muscle mass (r = 0.153) is evidence of its specificity for adiposity estimation, suggesting a lower dependence on lean mass than BMI [10,14]. This specificity allows CUN-BAE to address a fundamental limitation of BMI, providing a closer association with relative body fat percentage (r = 0.918 vs. 0.884 for BMI) [25], thereby improving the identification of conditions like normal weight obesity [5].

The Bland–Altman analysis further supports the usefulness of CUN-BAE for group-level estimation of body fat percentage; however, the observed limits of agreement indicate that BIA remains preferable when precise individual body composition assessment is required. Similarly, Endukuru et al. reported close agreement between CUN-BAE and BIA for body fat percentage, although their Bland–Altman analysis also suggested that individual-level and sex-specific differences should be considered [26].

### 4.2. Reaffirming the Distinct and Complementary Roles of BMI and CUN-BAE

Contrary to the notion that enhanced indices universally surpass it, our data reaffirm the enduring value of BMI. It demonstrated stronger bivariate correlations with both visceral fat (r = 0.941) and total fat mass in kilograms (r = 0.968) than CUN-BAE, confirming its role as a robust, simple surrogate for the absolute burden of metabolically active adipose tissue and for population-level risk stratification [3,4,5]. This strength, however, is counterbalanced by its inability to differentiate tissue type, as noted above. While BMI excels at quantifying absolute fat mass, our study sought to explore whether CUN-BAE could provide additional, refined insights into specific fat compartments, particularly visceral adipose tissue (VAT), whose quantification is paramount for metabolic risk stratification [8,11].

The sex-stratified analysis further supports the consistency of BMI and CUN-BAE across men and women, as both indices maintained strong correlations with adiposity parameters in each subgroup. However, the numerical differences in correlation values should be interpreted in light of known sex-related differences in body composition. Schorr et al. reported that, despite similar age and BMI, women had a higher percentage of fat mass and lower lean mass, whereas men had higher visceral adipose tissue and a more adverse cardiometabolic profile. These findings reinforce that BMI alone may not fully capture sex-specific differences in fat distribution and lean mass. Therefore, our results support the importance of considering sex when interpreting anthropometric and adiposity indices, particularly when these tools are used as indirect markers of visceral adiposity or body composition [27].

### 4.3. CUN-BAE as a Surrogate for Visceral Adiposity and the Modulating Role of Muscle Mass

We confirmed that CUN-BAE holds an independent association with VAT in adjusted models (β = 1.521), supporting its potential utility as a practical, non-imaging surrogate marker for this visceral adiposity. However, a crucial nuance emerged: this relationship was significantly influenced by skeletal muscle mass (β = −1.268) and ideal weight. Specifically, muscle mass showed a negative association in the adjusted model, suggesting that higher muscularity may affect the performance of CUN-BAE for estimating visceral adiposity. In contrast, ideal weight showed a positive association (β = 0.554), indicating that baseline body characteristics may influence the accuracy of this index for estimating this specific adipose compartment. Therefore, while CUN-BAE is a valuable indicator of visceral adiposity risk in the general population, this modulating effect refines its clinical application, highlighting that its optimal use for VAT estimation is context-dependent [11,25,28].

### 4.4. The Sarcopenia Paradox: Disconnect Between Muscle Quantity and Perceived Function

As a secondary exploratory outcome, SARC-F scores were low in this cohort and did not show meaningful variation across the distribution of adiposity and muscle mass measures. This observation is consistent with SARC-F being a screening tool that emphasizes perceived functional limitation and may have limited discriminatory capacity in relatively young or functionally preserved samples. These results should not be interpreted as evidence for or against sarcopenia; definitive evaluation would require objective measures of strength and performance in addition to muscle mass.

### 4.5. Clinical Implications and a Framework for Integrated Use

The evidence presented advocates for a shift from a competitive paradigm (CUN-BAE vs. BMI) to a complementary, sequential clinical framework that leverages the specific strengths of each tool. BMI should be retained as the universal, first-line screening tool due to its simplicity, strong epidemiological validation, and, as confirmed here, its robust correlation with absolute visceral and total fat mass. It serves as an excellent filter for identifying individuals with a high adiposity burden and associated cardiometabolic risk. The CUN-BAE index should be employed as a targeted, second-line assessment in specific clinical scenarios where BMI may be misleading or where a refined body composition analysis is warranted. These scenarios include: [1] evaluating patients with a normal BMI or those who are overweight and have components of metabolic syndrome in order to screen for normal weight obesity; [2] obtaining a more adiposity-oriented estimate for personalized nutritional, fitness, or surgical planning; and [3] serving as an accessible surrogate for visceral adiposity risk in non-athletic populations within primary care settings. For complex cases, such as suspected sarcopenic obesity, significant discrepancies between indices, or in situations in which precise monitoring is required, a third-line evaluation with direct body composition methods like bioelectrical impedance analysis (BIA) or dual-energy X-ray absorptiometry (DXA) is recommended to obtain definitive, compartmentalized data. This stratified approach optimizes resource utilization, enhances diagnostic precision, and facilitates personalized risk stratification and management.

### 4.6. Limitations and Future Directions

This study has limitations, including its cross-sectional design, which prevents the establishment of causal relationships between the indices and body composition parameters. Furthermore, while bioelectrical impedance analysis (BIA) provides a practical and validated assessment, the use of DEXA or computed tomography (CT) would serve as more definitive reference methods for fat distribution and visceral adipose tissue quantification. Despite these constraints, the study possesses notable strengths, including the consistent, strong correlations observed across multiple adiposity parameters, the detailed clinical and anthropometric characterization of the cohort, and the use of multivariate regression to control for key confounders, which strengthens the validity of the observed associations.

A specific and important limitation of the CUN-BAE index, as confirmed by our data, is its poor capacity for muscle mass assessment. This constraint is particularly relevant in the context of sarcopenic obesity, a high-risk phenotype characterized by the coexistence of high fat mass and low muscle mass and function. It is noteworthy that while our group with obesity displayed higher absolute muscle mass—a finding likely attributable to load-induced mechanical hypertrophy—this adaptive increase does not confer the same metabolic benefits as the lean mass associated with exercise and physical fitness. Adiposity-focused indices like CUN-BAE cannot differentiate between these qualitatively distinct types of muscle mass, which represents a fundamental constraint in their application for comprehensive body composition evaluation [29,30,31].

## 5. Conclusions

The CUN-BAE index is a robust and specific tool for estimating body fat percentage, effectively addressing the primary composition-related limitation of BMI. However, it also unexpectedly reaffirms the superior performance of BMI as an indicator of absolute visceral and total fat mass. Therefore, the clinical question should guide the choice of metric: BMI is superior for assessing the absolute burden of adiposity-related risk, while CUN-BAE is superior for evaluating the proportion of body weight that is fat. The Bland–Altman analysis supports the use of CUN-BAE for group-level estimation of body fat percentage, although BIA remains preferable when precise individual body composition assessment is required. Their integrated, context-driven use, complemented by direct body composition analysis when indicated, will enable a more precise and personalized approach to obesity assessment, metabolic risk stratification, and nutritional management.

## Figures and Tables

**Table 1:** Baseline characteristics of the study population (*n* = 204).

Characteristic	Value
**Demographics**	
Age (years)	41 ± 12
Weight (kg)	87.6 ± 27.5
Height (m)	1.63 ± 0.09
BMI (kg/m^2^)	32.9 ± 9.7
Female	65% (*n* = 133)
**Glycemic Status**	
Normal glycemia	71% (*n* = 145)
Prediabetes	25% (*n* = 50)
Type 2 diabetes	4% (*n* = 9)
**Comorbidities**	
Hypertension	25% (*n* = 50)
Hypothyroidism	19% (*n* = 38)
OSAHS	9% (*n* = 19)
Heart disease	7% (*n* = 14)
Pituitary adenoma	4% (*n* = 9)
Osteoporosis	3% (*n* = 6)
Immunological diseases	3% (*n* = 6)
Thyroid nodule	2% (*n* = 3)
Nephropathy	2% (*n* = 3)
COPD	2% (*n* = 3)
Hyperthyroidism	1% (*n* = 1)
MASLD	1% (*n* = 1)
Liver cirrhosis	1% (*n* = 1)
**Medication and Supplement Use**	
Any vitamins/supplements	43% (*n* = 68)
Vitamin D	20% (*n* = 41)
Other supplements	18% (*n* = 36)
Glucocorticoid use	1% (*n* = 1)
Testosterone use	1% (*n* = 2)
**Lifestyle habits**	
Alcohol use	31% (*n* = 63)
Drinks per month	7 ± 8
Smoking	13% (*n* = 16)
Smoking index (packs-years)	6 (IQR 1–6)
Drug use	2% (*n* = 3)
Marijuana use	100% of drug users (*n* = 3)
**Bioimpedance measurements**	
Muscle mass (kg)	28.8 ± 6.8
Total fat mass (kg)	35.8 ± 19.6
Total body fat mass (%)	38.5 ± 10.9
Visceral fat	13.5 ± 5.8

Data are presented as mean ± standard deviation, median (interquartile range IQR), or percentage (*n*). OSAHS: Obstructive Sleep Apnea–Hypopnea Syndrome; MASLD: Metabolically Associated Fatty Liver Disease; COPD: Chronic Obstructive Pulmonary Disease.

**Table 2:** Multiple regression analysis of BMI and CUN-BAE with adiposity parameters.

Dependent Variable	β Coefficient	95% CI	*p*
**CUN-BAE**			
Body fat percentage	0.882	0.809–0.955	<0.001
Women	0.822	0.715–0.929	<0.001
Men	0.716	0.615–0.817	<0.001
Total fat mass	0.433	0.369–0.496	<0.001
Women	0.460	0.405–0.516	<0.001
Men	0.411	0.355–0.467	<0.001
Visceral fat	1.521	1.136–1.679	<0.001
Women	1.171	0.984–1.359	<0.001
Men	1.097	0.937–1.257	<0.001
BMI			
Body fat percentage	0.604	0.530–0.679	<0.001
Women	0.847	0.760–0.935	<0.001
Men	0.619	0.510–0.727	<0.001
Total fat mass	0.461	0.439–0.484	<0.001
Women	0.513	0.486–0.540	<0.001
Men	0.405	0.371–0.439	<0.001
Visceral fat	1.080	0.960–1.20	<0.001
Women	1.012	0.846–1.178	<0.001
Men	0.832	0.622–1.043	<0.001

CI: confidence interval; BMI: body mass index; CUN-BAE: Clínica Universidad de Navarra-Body Adiposity Estimator. Models evaluating the association of CUN-BAE and BMI with body fat percentage and total fat mass were adjusted for glycemic status, hypertension, hypothyroidism, lung disease, smoking, alcohol, exercise, diet and nutritional counseling. Meanwhile, models evaluating the association of CUN-BAE and BMI with visceral fat percentage were adjusted for glycemic status, muscle mass, hypertension, lung disease, ideal weight, and diet.

## Data Availability

The data analyzed in the study are available from the corresponding author upon reasonable request.

## Abbreviations

The following abbreviations are used in this manuscript:

%BF	Body Fat Percentage
BIA	Bioelectrical Impedance Analysis
BMI	Body Mass Index
COPD	Chronic Obstructive Pulmonary Disease
CT	Computed Tomography
CUN-BAE	Clínica Universidad de Navarra-Body Adiposity Estimator
DEXA	Dual-Energy X-ray Absorptiometry
DXA	Dual-Energy X-ray Absorptiometry
IL-6	Interleukin 6
MASLD	Metabolically Associated Fatty Liver Disease
MRI	Magnetic Resonance Imaging
OSAHS	Obstructive Sleep Apnea–Hypopnea Syndrome
SARC-F	Strength, Assistance in Walking, Rising from a Chair, Climbing Stairs, Falls
SAT	Subcutaneous Adipose Tissue
SPSS	Statistical Package for the Social Sciences
T2D	Type 2 Diabetes
TNF-α	Tumor Necrosis Factor alpha
VAT	Visceral Adipose Tissue
WHO	World Health Organization
